# Comparison of the Nucleation Kinetics Obtained from the Cumulative Distributions of the Metastable Zone Width and Induction Time Data

**DOI:** 10.3390/molecules27093007

**Published:** 2022-05-07

**Authors:** Lie-Ding Shiau

**Affiliations:** 1 Department of Chemical and Materials Engineering, Chang Gung University, Taoyuan 333, Taiwan; shiau@mail.cgu.edu.tw; Tel.: +886-3-2118800 (ext. 5291); 2 Department of Urology, Chang Gung Memorial Hospital Linkou, Taoyuan 333, Taiwan

**Keywords:** crystallization, nucleation, interfacial energy, induction time, metastable zone width

## Abstract

A linearized integral model based on classical nucleation theory is applied in this work to determine the interfacial energy and pre-exponential factor using a linear plot from the cumulative distributions of the metastable zone width (MSZW) data for some systems reported in the literature, including isonicotinamide, butyl paraben, dicyandiamide, and salicylic acid. Based on the same criterion for the nucleation point, the interfacial energy and pre-exponential factor are determined using the conventional linear regression method from the cumulative distributions of the induction time data for the same systems. The results indicate that the interfacial energy and pre-exponential factor calculated from the MSZW data are consistent with those calculated from the induction time for the studied systems.

## 1. Introduction

Solute molecules can aggregate to form clusters in supersaturated solutions. Due to Ostwald ripening, the small clusters tend to dissolve while the large clusters continue to grow bigger. When the size of a cluster exceeds a critical size, it becomes thermodynamically stable, and this leads to the formation of a nucleus, referred to as nucleation [1,2,3]. During nucleation and the subsequent growth, the dynamics of liquid-crystalline phase separation plays an important role in the interfacial science [4,5]. In classical nucleation theory (CNT), the nucleation rate is expressed in the Arrhenius form governed by the interfacial energy and pre-exponential nucleation factor [1,2,3]. The interfacial energy is the energy required to create a new solid liquid interface for the formation of crystals in liquid solutions while the pre-exponential factor is related to the attachment rate of solute molecules to a cluster in the formation of crystals.

The induction time and the metastable zone width (MSZW) are two important measurements in determining the nucleation rate for a crystallization system. Due to the stochastic nature of crystal nucleation, a large variation in induction time and MSZW measurements for the appearance of a nucleus is observed in small volumes under the identical condition [6,7,8,9,10,11,12,13,14,15]. As the appearance of a nucleus in a supersaturated solution can be considered to be a random process by the Poisson’s law, Jiang and ter Horst [6] developed the cumulative induction time distributions, which can be applied to obtain the nucleation rate for each supersaturation. With the aid of the nucleation rates at different supersaturations, a single set of the interfacial energy and pre-exponential factor can then be determined based on CNT [6,10]. Later, Kadam et al. [8] proposed the cumulative MSZW distributions of detected nucleation events for a random process using the Poisson’s law, which can be applied to obtain the nucleation rate for each cooling rate. However, multiple sets of the interfacial energy and pre-exponential factor with a large variation among different cooling rates were determined using the probability distributions of the MSZW measurements [10]. Consequently, it is difficult to compare the interfacial energy and pre-exponential factor obtained from the cumulative induction time distributions with those obtained from the cumulative MSZW distributions.

As both the induction period and the MSZW of a crystallization system are directly related to the nucleation rate of the supersaturated solution, the same nucleation kinetics should be obtained on either the induction time data or the MSZW data for the same system [16,17]. Sangwal [18] related the nucleation rate with the rate of change of solution supersaturation at the MSZW limit on the number basis to recover the interfacial energy and pre-exponential factor from the MSZW. Xu et al. [19] modified the Sangwal’s theory to estimate the nucleation kinetic parameters of the eszopiclone-butyl acetate solution from the MSZW. However, the nucleation criterion of Sangwal’s theory for the MSZW has not been applied to determine the nucleation kinetic parameters from the induction time. Recently, Shiau [20] proposed a linearized integral model based on CNT to determine the interfacial energy and pre-exponential factor from the MSZW. In the present work, the linearized integral model developed by Shiau [20] is extended based on the appearance of a nucleus for the nucleation point to determine the interfacial energy and pre-exponential factor from the cumulative MSZW distributions for some systems reported in the literature. The results are compared with those determined based on the same nucleation criterion from the cumulative induction time distributions for the same systems.

## 2. Theoretical Derivations

The nucleation rate in solutions based on CNT is expressed as [1,2,3]

(1) $$J=A_{J}\;\exp\left[-\frac{16\pi \;v_{m}^{2}\;\gamma^{3}}{3k_{B}^{3}\;T^{3}\;\ln^{2}\;S}\right]$$

where $A_{J}$ is the nucleation pre-exponential factor, γ is the interfacial energy, $k_{B}$ is the Boltzmann constant, and $v_{m}=\frac{M_{w}}{\rho_{c}N_{A}}$ is the molecular volume.

The appearance of a nucleus in a supersaturated solution is often regarded as a random process [6,7,8,9,10,11,12,13,14,15]. The average number N(t) of expected nuclei generated from t=0 to a certain time t within a solution volume V is given by [8]

(2) $$N\left(t\right)=V\overset{t}{\underset{0}{\int }}J\left(t\right)\mathrm{dt}$$

where J is a function of the prevailing supersaturation and temperature.

As the appearance of a nucleus is regarded as a random process, the single nucleation mechanism has been proposed to relate the appearance of a nucleus with the detection of a nucleation point [6,7,8]. In the single nucleation mechanism, it is assumed that a single nucleus is formed at the nucleation time. This single nucleus grows to a certain size and then undergoes extensive secondary nucleation. The nucleation event is detected after the secondary nucleation of the single crystal. For simplicity, the growth tome between the formation of nuclei and their detection in form of crystals is assumed negligible [21]. Based on the single nucleation mechanisms, the induction time is defined as the time needed for a constant supersaturation operated at a given temperature from the establishment of the supersaturated state to the first appearance of a nucleus. As supersaturation is constant, J, is kept constant during the induction time period. The first appearance of a nucleus at the induction time $t_{i}$ corresponds to N(t)=1 for Equation (2), which reduces to [6]

(3) $$1={\mathrm{VJt}}_{i}$$

Due to the stochastic nature of the nucleation events, the first appearance of a nucleus at a certain time is usually described by a cumulative distribution function [6,7,8]. The median induction time $t_{i}$ is defined at 50% of fraction detected nucleation events from the cumulative distributions of the induction time data, which is the best predictor of a random variable to minimize the expected value of the absolute error [22].

Substituting Equation (1) into Equation (3) yields [23,24]

(4) $$\ln t_{i}=-\ln\left(A_{J}V\right)+\frac{16\pi \;\nu_{m}^{2}\;\gamma^{3}}{3k_{B}^{3}\;T^{3}\;\ln^{2}\;S}$$

Experimental induction time data can be evaluated at a given temperature by plotting $\ln t_{i}$ versus $\frac{1}{\ln^{2}\;S}$ for the determination of γ from the slope and $A_{J}$ from the intercept, respectively.

Similarly, based on the single nucleation mechanisms, the MSZW limit is defined as the time needed at a cooling rate from the establishment of the supersaturated state to the first appearance of a nucleus. As supersaturation increases during the cooling process, J starts from zero and increases during the MSZW period. The first appearance of a nucleus at the MSZW limit time $t_{m}$ corresponds to N(t)=1 for Equation (2), which reduces to

(5) $$1=V\overset{t_{m}}{\underset{0}{\int }}\mathrm{Jdt}$$

where $t_{m}$ represents the time at which the nucleation temperature $T_{m}$ is reached. Similarly, the median nucleation temperature $T_{m}$ is defined at 50% of fraction detected nucleation events from the cumulative distributions of the MSZW data [17,25,26]. Equation (5) is consistent with the cumulative MSZW distributions based on the first appearance of a nucleus adopted by Kulkarni et al. [10].

**Figure 1:**
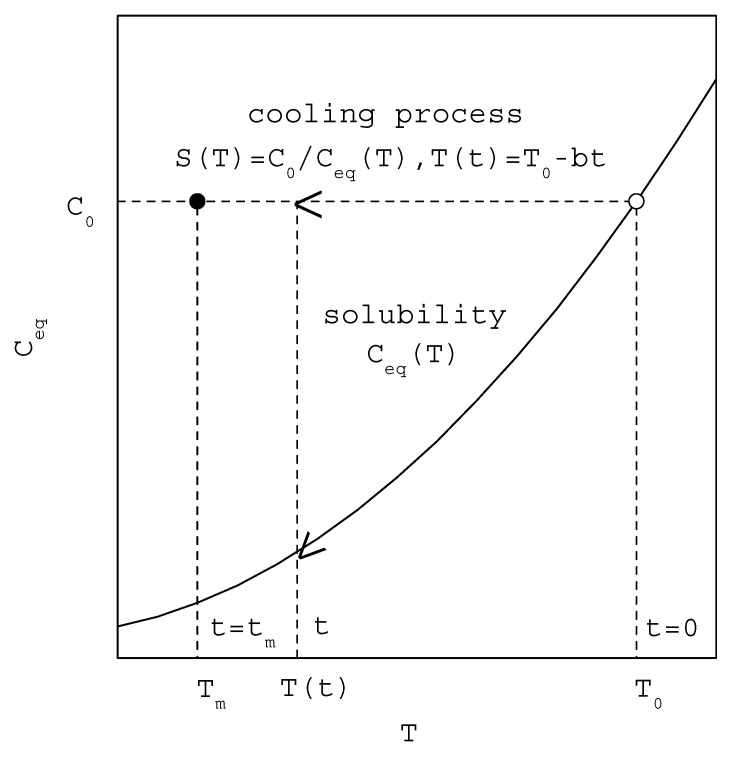
A schematic diagram [16] showing the increasing of supersaturation during the cooling process for the saturated concentration at $C_{0}$ (○ represents the starting point and ● represents the nucleation point at a given R).

As shown in Figure 1, $T_{0}$ is the initial saturated temperature at t=0, $T_{m}$ is the maximum undercooling temperature at $t_{m}$, ${\Delta T}_{m}=T_{0}-T_{m}$ is the MSZW, $C_{0}$ is the initial saturated concentration at $T_{0}$, and $S\left(T\right)=\frac{C_{0}}{C_{\mathrm{eq}}\left(T\right)}$ is the temperature-dependent supersaturation during the cooling process. Note that $C_{0}$ remains nearly unchanged in the MSZW. As temperature decreases during the cooling process, $C_{\mathrm{eq}}\left(T\right)$ usually decreases. Consequently, S(T) increases gradually from 1 at $C_{\mathrm{eq}}\left(T_{0}\right)=C_{0}$ during the cooling process. As defined in Equation (1), J starts from J=0 at t=0 and increases gradually as temperature decreases from $T_{0}$ to $T_{m}$.

As the nonlinear regression along with numerical integration involved in the integral model Equation (5) is complicated, Shiau [20] presented a linearized integral model to determine the nucleation kinetics from the MSZW data. Similarly, based on the two-point trapezoidal rule, Equation (5) leads to

(6) $$1=V\overset{t_{m}}{\underset{0}{\int }}\mathrm{Jdt}=\frac{1}{2}\left(J_{0}+J_{m}\right){\mathrm{Vt}}_{m}=\frac{J_{m}V\Delta T_{m}}{2b}$$

where $J_{0}$ and $J_{m}$ represent the nucleation rate at t=0 and $t=t_{m}$, respectively. Note that $J_{0}=0$ at t=0 when $S(T_{0})=1$, and $t_{m}=\Delta T_{m}/b$ for a constant cooling rate b.

According to Equation (1), the nucleation rate at $T_{m}$ is given by

(7) $$J_{m}=A_{J}\exp\left[-\frac{16{\pi v}_{m}{}^{2}\gamma^{3}}{3k_{B}{}^{3}T_{m}{}^{3}\ln^{2}S_{m}}\right]$$

where $S_{m}$ is the supersaturation at $T_{m}$ defined as $S_{m}=C_{0}/C_{\mathrm{eq}}\left(T_{m}\right)=C_{0}/C_{m}$. Substituting Equation (7) into Equation (6) yields

(8) $$\exp\left[-\frac{16{\pi v}_{m}{}^{2}\gamma^{3}}{3k_{B}{}^{3}T_{m}{}^{3}\ln^{2}S_{m}}\right]=\frac{2b}{A_{J}V\Delta T_{m}}$$

Taking the logarithm on both sides of Equation (8) gives

(9) $$\frac{1}{T_{m}{}^{3}\ln^{2}S_{m}}=\frac{3}{16\pi }\left(\frac{k_{B}{}^{3}}{v_{m}{}^{2}\gamma^{3}}\right)\left[\ln\left(\frac{\Delta T_{m}}{b}\right)+\ln\left(\frac{A_{J}V}{2}\right)\right]$$

If the temperature-dependent solubility is described in terms of the van’t Hoff equation [1], one obtains

(10) $$\ln S_{m}=\ln\left(\frac{C_{0}}{C_{m}}\right)=\frac{-\Delta H_{d}}{R_{G}}\left(\frac{1}{T_{0}}-\frac{1}{T_{m}}\right)=\left(\frac{\Delta H_{d}}{R_{G}T_{0}}\right)\left(\frac{\Delta T_{m}}{T_{m}}\right)$$

where $�H_{d}$ is the van’t Hoff heat of dissolution and $R_{G}$ is the ideal gas constant. Substituting Equation (10) into Equation (9) yields

(11) $${\left(\frac{T_{0}}{\Delta T_{m}}\right)}^{2}=\frac{3}{16\pi }{\left(\frac{k_{B}T_{0}}{v_{m}{}^{2/3}\gamma }\right)}^{3}{\left(\frac{\Delta H_{d}}{R_{G}T_{0}}\right)}^{2}\left[\ln\left(\frac{\Delta T_{m}}{b}\right)+\ln\left(\frac{A_{J}V}{2}\right)\right]$$

A plot of ${\left(\frac{T_{0}}{\Delta T_{m}}\right)}^{2}$ versus $\ln\left(\frac{\Delta T_{m}}{b}\right)$ based on the MSZW data at a given $T_{0}$ should give a straight line, the slope and intercept of which allow to determine γ and $A_{J}$, respectively.

Thus, both Equation (11) for the MSZW and Equation (4) for the induction time are originally derived from Equation (2). In other words, the same nucleation criterion is adopted to determine γ and $A_{J}$ using both Equation (11) for the MSZW and Equation (4) for the induction time. Consequently, γ and $A_{J}$ obtained using Equation (11) from the MSZW can be compared to those obtained using Equation (4) from the induction time for the same system.

## 3. Results and Discussions

The MSZW data are usually experimentally measured by cooling a supersaturated solution at a constant cooling rate from the initial saturated temperature $T_{0}$. The temperature measured at the nucleation point is defined as $T_{m}$ and ${\Delta T}_{m}=T_{0}-T_{m}$ is the MSZW. Note that the heat of crystallization is usually small and is quickly removed by the cooling medium as the MSZW experiments are operated at a controlled cooling rate [1]. The cumulative distributions of the MSZW data are analyzed using Equation (11) for some systems reported in the literature, including isonicotinamide (INA) [10], butyl paraben (BP) [26], dicyandiamide (DCD) [17], and salicylic acid (SA) [25]. The obtained results of γ and $A_{J}$ from the MSZW data are compared with those from the induction time data reported in the literature.

**Figure 2:**
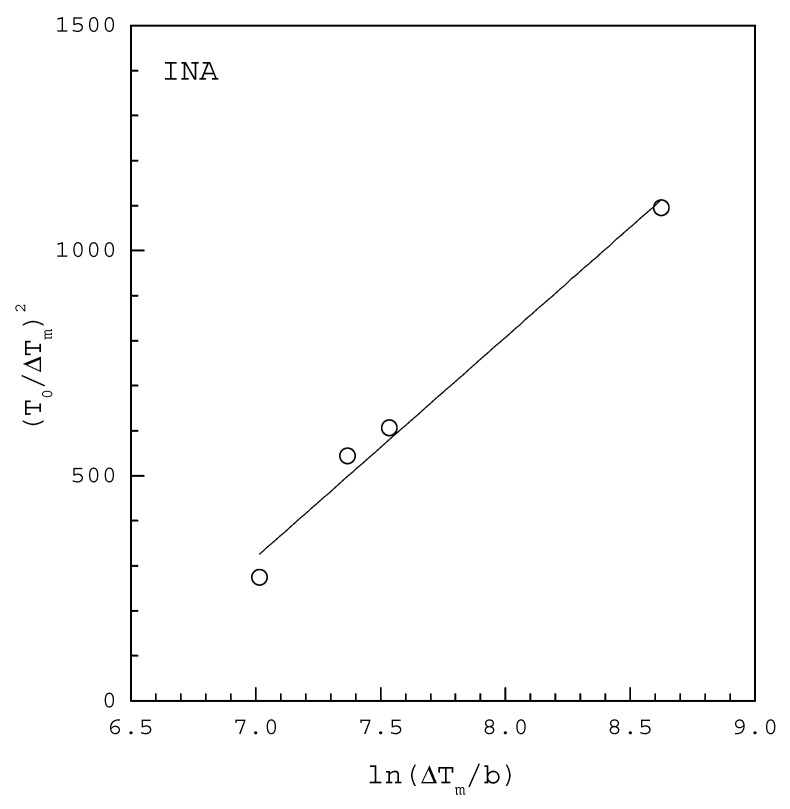
The MSZW data fitted to the linearized Equation (11) for INA in 1 mL ethanol saturated at $T_{0}=307.76\;K$, where ${\Delta T}_{m}$ for each b is extracted at 50% of fraction detected nucleation events from the cumulative distributions of the MSZW data obtained by Kulkarni et al. [10].

Figure 2 shows the MSZW data fitted to the linearized Equation (11) for INA in 1 mL ethanol saturated at $T_{0}=307.76\;K$, where ${\Delta T}_{m}$ for each b is extracted at 50% of fraction detected nucleation events from the cumulative distributions of the MSZW data obtained by Kulkarni et al. [10]. The fitting of Equation (11) leads to $\gamma =2.96\;\mathrm{mJ}/m^{2}$ and $A_{J}=3499\;m^{-3}\;s^{-1}$ with $R^{2}=0.975$ in Table 1. Note that $v_{m}=1.685\times 10^{-28}\;m^{3}$ for INA. According to the solubility reported by Kulkarni et al. [10], $\Delta H_{d}=22.7\;\mathrm{kJ}/\mathrm{mol}$ is used for the van’t Hoff solubility equation. For comparison, Table 1 also lists $\gamma =3.60\;\mathrm{mJ}/m^{2}$ and $A_{J}=6600\;m^{-3}\;s^{-1}$ reported by Kulkarni et al. [10] for INA in 1 mL ethanol using the cumulative distributions of the induction time data fitted to Equation (4).

**Figure 3:**
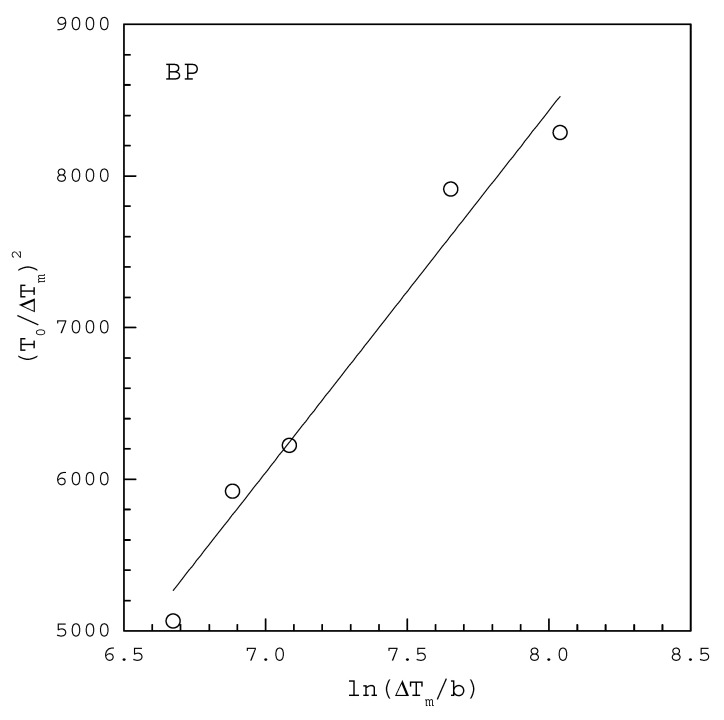
The MSZW data fitted to the linearized Equation (11) for BP in 5 mL ethanol saturated at $T_{0}=313.15\;K$, where ${\Delta T}_{m}$ for each b is extracted at 50% of fraction detected nucleation events from the cumulative distributions of the MSZW data obtained by Yang [26].

Figure 3 shows the MSZW data fitted to the linearized Equation (11) for BP in 5 mL ethanol saturated at $T_{0}=313.15\;K$, where ${\Delta T}_{m}$ for each b is extracted at 50% of fraction detected nucleation events from the cumulative distributions of the MSZW data obtained by Yang [26]. The fitting of Equation (11) leads to $\gamma =0.86\;\mathrm{mJ}/m^{2}$ and $A_{J}=4588\;m^{-3}\;s^{-1}$ with $R^{2}=0.961$ in Table 1. Note that $v_{m}=2.57\times 10^{-28}\;m^{3}$ for INA. According to the solubility reported by Yang and Rasmuson [27], $\Delta H_{d}=11.9\;\mathrm{kJ}/\mathrm{mol}$ is used for the van’t Hoff solubility equation. For comparison, Table 1 also lists $\gamma =1.15\;\mathrm{mJ}/m^{2}$ and $A_{J}=2752\;m^{-3}\;s^{-1}$ reported by Yang and Rasmuson [23] for BP in 5 mL ethanol using the cumulative distributions of the induction time data fitted to Equation (4).

**Figure 4:**
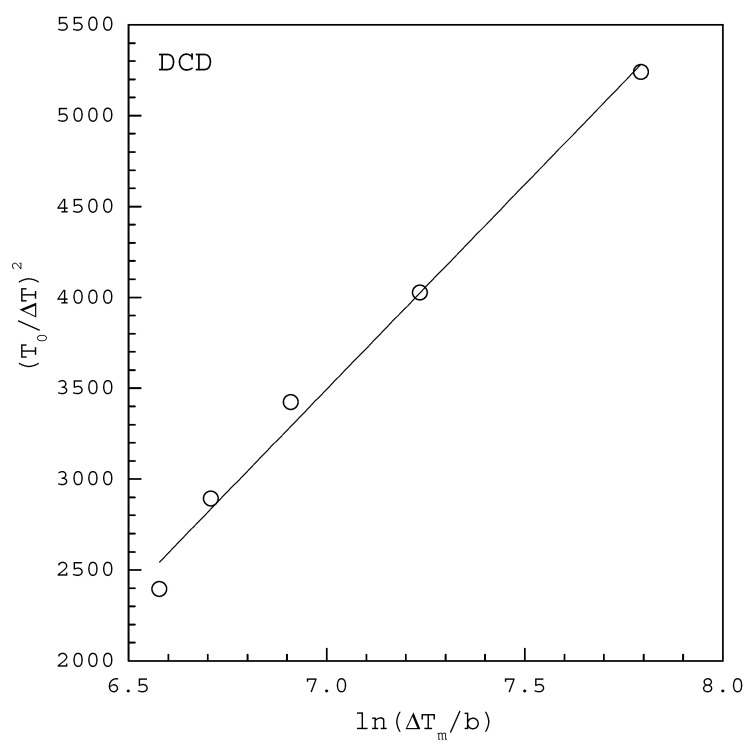
The MSZW data fitted to the linearized Equation (11) for DCD in 100 mL water saturated at $T_{0}=293.15\;K$, where ${\Delta T}_{m}$ for each b is extracted at 50% of fraction detected nucleation events from the cumulative distributions of the MSZW data obtained by Si et al. [17].

Figure 4 shows the MSZW data fitted to the linearized Equation (11) for DCD in 100 mL water saturated at $T_{0}=293.15\;K$, where ${\Delta T}_{m}$ for each b is extracted at 50% of fraction detected nucleation events from the cumulative distributions of the MSZW data obtained by Si et al. [17]. The fitting of Equation (11) leads to $\gamma =3.24\;\mathrm{mJ}/m^{2}$ and $A_{J}=86\;m^{-3}\;s^{-1}$ with $R^{2}=0.988$ in Table 1. Note that $v_{m}=9.52\times 10^{-29}\;m^{3}$ for DCD. According to the solubility reported by Zhang et al. [28], $\Delta H_{d}=32.4\;\mathrm{kJ}/\mathrm{mol}$ is used for the van’t Hoff solubility equation. For comparison, Table 1 also lists $\gamma =2.77\;\mathrm{mJ}/m^{2}$ and $A_{J}=58\;m^{-3}\;s^{-1}$ reported by Si et al. [17] for DCD in 100 mL water using the cumulative distributions of the induction time data fitted to Equation (4).

**Figure 5:**
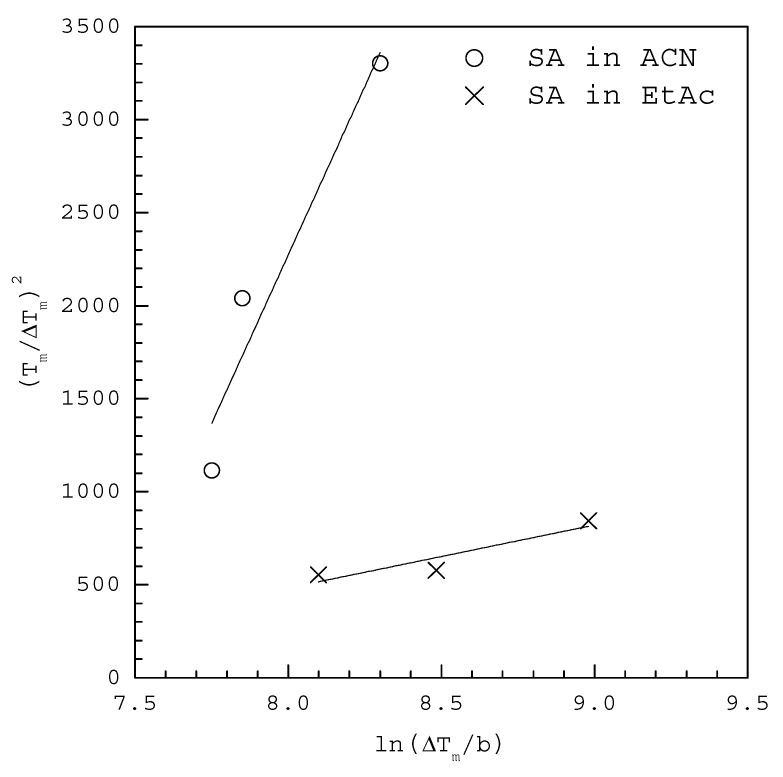
The MSZW data fitted to the linearized Equation (11) for SA in 20 mL ACN and EtAc saturated at $T_{0}=323.15\;K$, where ${\Delta T}_{m}$ for each b is extracted at 50% of fraction detected nucleation events from the cumulative distributions of the MSZW data obtained by Mealey et al. [25].

Figure 5 shows the MSZW data fitted to the linearized Equation (11) for SA in 20 mL acetonitrile (ACN) and ethyl acetate (EtAc) saturated at $T_{0}=323.15\;K$, where ${\Delta T}_{m}$ for each b is extracted at 50% of fraction detected nucleation events from the cumulative distributions of the MSZW data obtained by Mealey et al. [25]. The fitting of Equation (11) leads to $\gamma =1.62\;\mathrm{mJ}/m^{2}$ and $A_{J}=63\;m^{-3}\;s^{-1}$ with $R^{2}=0.935$ in ACN and $\gamma =2.26\;\mathrm{mJ}/m^{2}$ and $A_{J}=140\;m^{-3}\;s^{-1}$ with $R^{2}=0.866$ in EtAc in Table 1. Note that $v_{m}=1.59\times 10^{-28}\;m^{3}$ for SA. According to the solubility reported by Nordstrom and Rasmuson [29], $\Delta H_{d}=23.0\;\mathrm{kJ}/\mathrm{mol}$ in ACN and $\Delta H_{d}=11.6\;\mathrm{kJ}/\mathrm{mol}$ in EtAc are used for the van’t Hoff solubility equations. For comparison, Table 1 also lists $\gamma =1.71\;\mathrm{mJ}/m^{2}$ and $A_{J}=285\;m^{-3}\;s^{-1}$ in ACN and $\gamma =2.03\;\mathrm{mJ}/m^{2}$ and $A_{J}=144\;m^{-3}\;s^{-1}$ in EtAc reported by Kakkar et al. [24] for SA using the cumulative distributions of the induction time data fitted to Equation (4).

As indicated in Table 1, it is concluded that γ and $A_{J}$ calculated from the cumulative distributions of the MSZW data using Equation (11) are consistent with calculated those from the cumulative distributions of the induction time data using Equation (4) for the studied systems. It should be noted that, as the appearance of a nucleus is regarded as a random process, both the measured MSZW and induction time data under each condition usually exhibit a distribution of value instead of a certain value [6,7,8]. Consequently, the fitting in Figure 3, Figure 4 and Figure 5 is considered quite satisfactory for the scattered experimental MSZW data due to the stochastic nature of the nucleation events.

## 4. Conclusions

A linearized integral model based on CNT is developed in this work to determine the interfacial energy and pre-exponential factor using a linear plot of ${\left(\frac{T_{0}}{\Delta T_{m}}\right)}^{2}$ versus $\ln\left(\frac{\Delta T_{m}}{b}\right)$ from the cumulative distributions of the MSZW data for some systems reported in the literature, including isonicotinamide, butyl paraben, dicyandiamide, and salicylic acid. The results indicate that the interfacial energy and pre-exponential factor obtained from the MSZW are consistent with those obtained using a linear plot of $\ln t_{i}$ versus $\frac{1}{\ln^{2}\;S}$ from the cumulative distributions of the induction time data based on the same criterion for the nucleation point for the same systems. It is validated that, as both the induction time and the MSZW of a crystallization system are directly related to the nucleation rate of the supersaturated solution, the same nucleation kinetics are obtained based on either the induction time data or the MSZW data for the same system. The unique feature of this work is that the developed novel linearized integral model provides a simple method to determine the interfacial energy and pre-exponential factor from the cumulative MSZW distributions based on the first appearance of a nucleus at the nucleation point.

## Figures and Tables

**Table 1:** Comparison of γ and $A_{J}$ obtained from the MSZW and induction time data for some crystallization systems.

	MSZW			$t_{i}$	
	$\gamma \;\left(\mathrm{mJ}/m^{2}\right)$	$A_{J}\;\left(m^{-3}\;s^{-1}\right)$	$R^{2}$	$\gamma \;\left(\mathrm{mJ}/m^{2}\right)$	$A_{J}\;\left(m^{-3}\;s^{-1}\right)$
INA	2.96	3499	0.975	3.60	6600
BP	0.86	4588	0.961	1.15	2752
DCD	3.24	86	0.988	2.77	58
SA in ACN	1.62	63	0.935	1.71	285
SA in EtAc	2.26	140	0.866	2.03	144

## Data Availability

Data is contained within the article.

## Nomenclature

Notation	
$A_{J}$	pre-exponential nucleation factor $\left(m^{-3}\;s^{-1}\right)$
b	cooling rate (K/s)
$C_{0}$	initial concentration of solute molecules $\left(m^{-3}\right)$
$C_{\mathrm{eq}}$	equilibrium concentration of solute molecules $\left(m^{-3}\right)$
J	nucleation rate $\left(m^{-3}\;s^{-1}\right)$
$k_{B}$	Boltzmann constant $\left(=1.38\times 10^{-23}\;J/K\right)$
$M_{W}$	molar mass (kg/mol)
$N_{A}$	Avogadro number $\left(=6.02\times 10^{23}\;{\mathrm{mol}}^{-1}\right)$
$R_{G}$	gas constant $\left(=8.314\;J\;{\mathrm{mole}}^{-1}\;K^{-1}\right)$
S	supersaturation ratio, (−)
T	temperature (K)
$T_{0}$	initial saturated temperature for the initial saturated solute concentration (K)
$T_{m}$	temperature at the MSZW limit at $t_{m}\left(K\right)$
t	time (s)
$t_{i}$	induction time (s)
$t_{m}$	time at the MSZW limit (s)
V	solution volume $\left(m^{3}\right)$
$v_{m}$	volume of the solute molecule $\left(m^{3}\right)$
Greek letters	
γ	interfacial energy (J/m^2^)
$\rho_{C}$	crystal density (kg/m^3^)
${\Delta T}_{m}$	MSZW (K).
